# Implanted in the Scar: A Case Report of Diagnosis and Management of Cesarean Scar Ectopic Pregnancy

**DOI:** 10.5811/cpcem.47265

**Published:** 2025-12-10

**Authors:** Hanna Schindler, Leila Keeler

**Affiliations:** Orlando College of Osteopathic Medicine, Winter Garden, Florida

**Keywords:** ectopic pregnancy, cesarean scar ectopic pregnancy, maternal morbidity and mortality, transvaginal ultrasound, women’s health

## Abstract

**Introduction:**

Ectopic pregnancy is a serious pregnancy complication that occurs when a gestational sac implants outside the uterus, most commonly in the fallopian tubes. However, a rare form of ectopic pregnancy, the cesarean scar ectopic pregnancy, occurs within a prior cesarean section scar and is becoming more common as cesarean delivery rates continue to rise. Cesarean scar ectopic pregnancies are challenging to diagnose and pose significant risks, including rupture and hemorrhage, which can lead to maternal death.

**Case Report:**

A 27-year-old woman presented to the emergency department with a 16-day history of abdominal pain and vaginal bleeding, initially believed to be her menstrual period. She had a history of one previous lower uterine segment cesarean section. On examination, her beta-human chorionic gonadotropin (β-hCG) levels were elevated, and transvaginal ultrasound revealed an empty uterus with a gestational sac within a cystic area of the cesarean scar. The patient was diagnosed with a cesarean scar ectopic pregnancy. Given the high rupture risk, she underwent laparoscopic surgery with dilation and curettage. Postoperative management included methotrexate, antibiotics, and analgesics. A follow-up β-hCG test showed a significant decline, confirming resolution of the ectopic pregnancy. At her two-week follow-up, the patient remained asymptomatic with no bleeding, and ultrasound confirmed no retained products of conception.

**Conclusion:**

Cesarean scar ectopic pregnancies are a rare and life-threatening complication of pregnancy that require timely diagnosis and intervention. Early detection through transvaginal ultrasound and appropriate multidisciplinary management are critical to prevent adverse outcomes. This case highlights the importance of early recognition, classification, surgical decision-making, and standardized diagnostic protocols to improve outcomes and save lives.

## INTRODUCTION

An ectopic pregnancy occurs when a gestational sac implants outside the uterus; it is a known complication of pregnancy. This complication can present with vaginal bleeding and other non-specific and variable symptoms such as lower abdominal pain, nausea, and vomiting. Women in their first trimester who present to the emergency department (ED) with this complication have a prevalence for ectopic pregnancy of 18%. It is easily misdiagnosed, sharing symptomatology with appendicitis, urinary calculi, early pregnancy loss, or trauma.^1^,^2^ Due to this symptomatic mimicry and resultant misdiagnosis, ectopic pregnancies are the leading cause of maternal mortality in the first trimester, with an incidence of 5–10% of all pregnancy-related deaths.^2^,^3^

Approximately 90% of ectopic pregnancies are implanted within the ampulla of the fallopian tube; however, a rare form occurs when a gestational sac implants in a previous cesarean scar. Cesarean scar ectopic pregnancies occur in 4% of all ectopic pregnancies, as well as 1 in 2000 pregnancies in women who underwent at least one prior cesarean section.^4^ With the rate of cesarean deliveries steadily increasing and improved detection technology through sonographic imaging, the incidence of cesarean scar ectopic pregnancies has also risen.^5^

In this case report we highlight a challenging diagnostic situation that clinicians may encounter, which requires a high degree of clinical suspicion and often can require urgency. Cesarean scar ectopic pregnancies can present subtly and carry a high risk for uterine rupture and catastrophic hemorrhage, making early recognition critical in the emergency setting. This case report highlights key symptomatic features that support early diagnosis and guideline-based classification of cesarean scar ectopic pregnancy. It also emphasizes the value of clinical–imaging correlation in guiding treatment decisions, as well as the resulting management outcomes.

## CASE REPORT

A 27-year-old female presented to the ED with a 16-day history of abdominal pain and vaginal bleeding, which she initially believed to be her menstrual period. Three months earlier, she had undergone a lower uterine segment cesarean section for fetal distress. Her post-surgical bleeding had resolved approximately two months prior, but the onset of these new symptoms prompted her to seek medical attention.

The patient’s full obstetric history included six total previous pregnancies, three term births, two vaginal deliveries, one lower transverse cesarean section previously disclosed, and two abortions. Her last menstrual period had been reported approximately one month prior to her presentation to the ED. She had no history of chronic medical illnesses and was not taking any medications at the time of her ED arrival.

General physical exam was normal with the only pertinent positive being suprapubic and lower left quadrant abdominal tenderness. Her blood pressure was 119/74 millimeters of mercury (mmHg), and her temperature was 37 °C. On admission, her beta-human chorionic gonadotropin (β-hCG) level was 9,623 international units per milliliter (IU/mL) (reference range: < 5 IU/L), and after admission, a retest 48 hours later, per the standard guideline set by the American College of Emergency Physicians (ACEP), showed the β-hCG value was 16,587 IU/mL, an increase of over 66%, consistent with early pregnancy progression. A transvaginal ultrasound revealed an empty uterine cavity, and a possible gestational sac within a cystic region of the cesarean section scar, near the endometrium. At this point, the patient was admitted for treatment of an ectopic pregnancy, likely within a previous cesarean section scar.

Treatment options of oral methotrexate and surgical resection were considered, and risks and benefits were discussed with the patient. Surgical resection was recommended due to the high risk of rupture and hemorrhage based on the location of the ectopic pregnancy. The patient opted for laparoscopic and transvaginal ultrasound-guided removal via dilation and curettage. The patient underwent general anesthesia and sterile vaginal preparation. Local anesthesia was administered, followed by an infraumbilical incision. A secondary 5-mm trocar was inserted for visualization.

*CPC-EM Capsule*

What do we already know about this clinical entity?

*Cesarean scar ectopic pregnancy is a rare but increasingly recognized complication in patients with prior cesarean delivery, carrying a significant risk of uterine rupture.*

What makes this presentation of disease reportable?

*Subtle symptoms in a stable patient with a history of cesarean delivery led to early diagnosis and coordinated multidisciplinary care.*

What is the major learning point?

*Early recognition, classification, and prompt intervention are vital for reducing risk of rupture and maternal morbidity and mortality.*

How might this improve emergency medicine practice?

*Clinicians should have a heightened awareness in post-cesarean patients with abdominal pain or bleeding, emphasizing early ultrasound and multidisciplinary evaluation.*

**Image 1:**
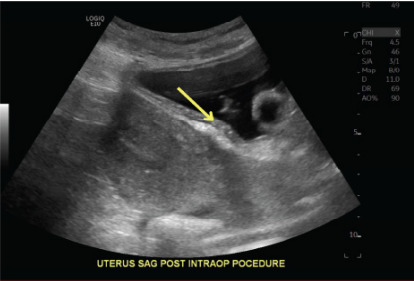
Transvaginal ultrasound showing uterus after products of conception were removed (arrow).

Upon laparoscopic visualization, dense adhesions were noted between the omentum and uterine fundus, as well as between the anterior peritoneal reflection and the lower uterine segment, likely sequelae of a prior cesarean section, and a known risk factor for cesarean scar ectopic pregnancy. Intraoperative transabdominal ultrasound confirmed a gestational sac implanted within the cesarean scar. Under ultrasound guidance the cervix was dilated, and a suction curette was introduced into the scar site. A total of eight suction passes were performed until transvaginal ultrasound confirmed evacuation of all products of conception (Image 1). Hemostasis was achieved, and laparoscopy confirmed no evidence of uterine perforation. Fascia and skin were closed in layers with absorbable sutures, and the incision was sealed with Dermabond™ (Ethicon, Inc, Somerville, NJ).

Postoperatively, the patient was given one dose of methotrexate and started on doxycycline 100 mg by mouth once daily for five days, methylergonovine maleate 0.2 mg by mouth every six hours for one day, and hydrocodone-acetaminophen, every six hours as needed for moderate pain for three days. The patient was also instructed to continue her combined oral contraceptive pill (ethinyl estradiol/norethindrone/ferrous fumarate) daily and cephalexin 500 mg by mouth four times daily for 10 days, which was previously started in the ED. A follow-up β-hCG test showed a significant decline, confirming resolution of the ectopic pregnancy. At her two-week follow-up, the patient remained asymptomatic with no further vaginal bleeding. Ultrasound imaging confirmed the absence of retained products of conception, and the β-hCG was negative at this time.

## DISCUSSION

An ectopic pregnancy was considered among the differential diagnoses, alongside miscarriage, when the patient presented to the ED with an initial β-hCG level of 9,623 IU/mL. At that time the patient was admitted. A repeat β-hCG measurement, 48 hours later, showed an increase to 16,587 IU/L, representing a rise of more than 66%, which is consistent with the expected increase seen in a normal early pregnancy. However, transvaginal ultrasound showed an empty uterus despite the patient’s β-hCG level being well above the discriminatory zone of 1,500–2000 milli-IU/mL, at which point gestational structures on transvaginal ultrasound are typically visible. These combined findings are what prompted the diagnosis of ectopic pregnancy.^7^

**Image 2:**
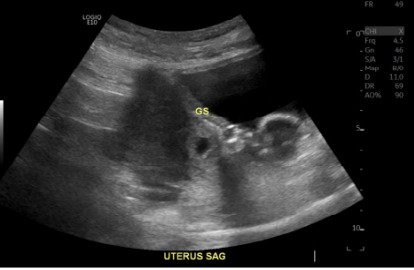
Sagittal view of the uterus demonstrating a gestational sac visualized in the lower uterine cesarean scar with progression toward the uterine cavity. *GS*, gestational sac; *uterus SAG*, uterine prolapse.

Serial β-hCG measurements are a valuable tool in assessing early pregnancy, as the rate of rise can inform clinical decision-making. Typically, a rise of at least 35–66% over 48 hours is expected in a viable intrauterine pregnancy. However, a plateau or suboptimal rise may suggest a nonviable pregnancy or ectopic pregnancy, although it is not confirmatory and transvaginal ultrasound is also needed. According to current clinical guidelines, including those from ACEP and the American College of Obstetricians and Gynecologists, serial β-hCG measurements should always be interpreted in conjunction with transvaginal ultrasound findings to determine pregnancy location and viability. A cesarean scar ectopic pregnancy was ultimately diagnosed after a hyperechoic, cyst-like structure was visualized within the cesarean scar tissue (Image 2) without anechoic or echogenic free fluid present in the pouch of Douglas.^7^,^8^

Cesarean scar ectopic pregnancies head special consideration through typing based on the site of implantation. Type 1 occurs when the gestational sac develops in the myometrium and grows toward the cervico-isthmic space or uterine cavity, while type 2 occurs when the gestational sac grows toward the bladder and abdominal cavity.^8^ In the case of this patient, type 1 classification was determined due to the nature of ectopic positioning within the scar, growing toward the uterine cavity. Rupture and hemorrhage potential is of great concern in cesarean scar ectopic pregnancies due to increased vasculature within the uterine segment; thus, in the case of our patient, uterine rupture potential increased the emergent nature of her ectopic pregnancy presentation.^9^ Additionally, due to the β-hCG elevation > 5,000 IU/mL, methotrexate monotherapy was not an appropriate expectant management based on increased possibility of treatment failure at these levels in addition to the high risk of rupture due to positioning.^7^

Intrauterine excision and methotrexate as combination therapy was determined to be the necessary treatment modality. Dilation and curettage of the gestational sac with laparoscopic uterine visualization was indicated based on type-1 positioning and the patient’s good hemodynamic stability. The laparoscope was used to ensure the gestational sac did not breach the full width of the myometrium or the serosa of the uterus and for visualization during dilation and curettage to ensure no perforation occurred. If the gestational sac had perforated the uterine wall, laparoscopic removal or laparotomy could have been used for removal and to control intra-abdominal bleeding. Additionally, laparoscopic intervention and removal can be beneficial for reduction in recurrence due to the ability for scar revision by removal and reclosure of the scar tissue area; however, recurrence potential is still present, especially in patients without scar revision. Patients should be informed of potential risk and serious sequelae of a recurrence of cesarean scar ectopic pregnancy.^10^

## CONCLUSION

Cesarean scar ectopic pregnancies are a rare, life-threatening maternal risk and a clinically challenging diagnosis. Given their non-specific presentation, ranging from mild abdominal pain to vaginal bleeding, a missed diagnosis could lead to delayed treatment resulting in rupture, hemorrhage, and maternal death. A training protocol that includes a multidisciplinary team approach consisting of emergency medicine, obstetrics and gynecology, radiology, and surgical specialists when needed can facilitate timely diagnosis and allow for earlier treatment and better patient outcomes. Early evaluation and point-of-care ultrasound should be considered with heightened suspicion of cesarean scar ectopic pregnancy with a presentation of fitting symptomatology and history of a previous cesarean delivery. Especially in resource-limited settings, where access to advanced imaging and specialist consultation may be delayed, early point-of-care assessment and rapid response for available treatment could be life-saving.
